# Healthy Team Healthy U: A Prospective Validation of an Evidence-Based Worksite Health Promotion and Wellness Platform

**DOI:** 10.3389/fpubh.2015.00188

**Published:** 2015-08-04

**Authors:** Linn Goldberg, Chondra Lockwood, Bharti Garg, Kerry S. Kuehl

**Affiliations:** ^1^Department of Medicine, Division of Health Promotion and Sports Medicine, Oregon Health & Science University, Portland, OR, USA

**Keywords:** health promotion, wellness, employee health, occupational health, physical activity, diet, body mass index, blood pressure

## Abstract

**Objective:**

To evaluate the effects of a research-tested, team-based health promotion and wellness program combined with digital technologies and implemented in a diverse worksite setting among hospital, clinic, and university employees.

**Methods:**

A prospective cohort study of employees completing biometrics and questionnaires before and after the initial 12-session wellness program and its 12-session booster, 1 year later.

**Results:**

After both the initial intervention and booster, blood pressure and weight were reduced, with greater reductions among employees with pre-hypertension and hypertension and those with a body mass index ≥25. After both the initial intervention and booster, there was a significant increase in (1) daily intake of fruit and vegetable servings, (2) days/week of ≥30 min of exercise, (3) days/week of strength training, and (4) levels of moderately vigorous and vigorous daily physical activity. Self-reported indices of both depression and work-related stress were reduced, while participants reported increased happiness. Post booster, average sleep quality, and sleep duration increased among higher risk employees reporting ≤6 h of daily sleep. Employees reported receiving encouragement from co-workers to engage in healthful diet and physical activities, and exercised together more, and indicated that they would recommend the program to other employees. Longitudinal analysis revealed the durability of the initial intervention outcomes with further beneficial effects after the booster.

**Conclusion:**

A research tested, comprehensive team-based health promotion and wellness program, combined with digital technologies, improved employee health behaviors, mood, sleep, elements of co-worker cohesion, and biometrics among a diverse multi-site workforce. Positive program effects were durable, with enhanced results after the booster.

## Introduction

The majority of U.S. health care costs are due to conditions related to unhealthy behaviors and their associated consequences (1–4). During the past decade, workers and employers have experienced an 80% increase in total premiums without the benefit of improved health outcomes (5). Recognizing that effective health promotion and wellness programs may be a low-cost solution to change unhealthy behaviors, improving employee health, and lower rising medical expenditures, the National Institute of Occupational Safety and Health and the U.S. government’s Affordable Care Act support use of health promotion initiatives in the workplace (6, 7). Although occupational settings have the potential to provide programs that lead to beneficial health outcomes, few commercialized wellness programs have documented effectiveness, and fewer evidence-based programs are available for commercial use (8).

Improving health behaviors have the potential to impact employee health, safety, and productivity, as well as reduce employee and employer direct and indirect costs. For example, overweight and obese employees contribute to higher medical and operating costs due to more absenteeism and presenteeism, and greater medical expenditures related to higher body mass indices (9, 10). Employees with pre-hypertension or hypertension report higher levels of stress and greater absenteeism (11, 12). Furthermore, previous research has demonstrated that higher workplace costs are related to depression, higher stress, and inadequate sleep quality and quantity (13–17).

Workplace wellness programs can benefit both workers and employers by targeting healthy behaviors and certain medical conditions. Interventions that increase fruit and vegetable intake and enhance physical activity (PA) have been shown to lower health care expenditures (18). Fruit and vegetable intake of five servings/day is associated with a lower risk of chronic medical illnesses, including cardiovascular disease, type 2 diabetes, and certain cancers (18–22). Likewise, achieving even low levels of regular PA among those who are sedentary can attenuate health effects of obesity and reduce blood pressure (23, 24).

The wellness intervention assessed is Healthy Team Healthy U™ (HTHU), a team based health and safety intervention paradigm developed by the Division of Health Promotion and Sports Medicine at the Oregon Health & Science University. The intervention includes a research-tested behavior change program based on our previous randomized controlled clinical trials (25–37). The program comprises an interactive curriculum targeting specific health, exercise, and nutrition topics with theoretical underpinnings influenced by the Social Learning Theory and Theory of Reasoned Action and its modification, the Theory of Planned Behavior (38–40). To enhance workplace translation and scalability for employees located at multiple sites, we combined our prior team-based intervention paradigm with a web-based digital platform in order to (1) facilitate increased interaction, communication, and peer support, (2) improve participants’ activity tracking and monitoring capabilities, and (3) deliver digital resources to participants.

The prior research-studied models were implemented with small teams of three to seven participants, self-administered by team members. The intervention paradigm has demonstrated numerous benefits, including enhanced health knowledge (25–28, 33), improved dietary practices (25, 28–32), greater exercise self-efficacy and PA (25, 29, 31, 32), and higher measured fitness, as assessed by both maximal oxygen uptake (VO_2_ max) and muscular endurance (27, 29, 32). Other positive outcomes have included maintaining or achieving a healthier body mass index (BMI) (kilogram body weight per square millimeter) (27, 29), reduced LDL-cholesterol (29), decreased drug and alcohol use (26, 28, 33), lower levels of drinking and driving (31), improved sleep quantity and quality (30), reduced injuries (27, 28, 30, 34), less workman compensation claims, and lower health care costs (34). Beyond indices of physical health and reduced expenditures, the intervention model has improved mood (26) and worker cohesion (27, 29), reduced personal stress (30) and resulted in a higher perception of wellbeing (27, 37). The mechanism of the model’s programmatic effects have been delineated by deconstructing its processes using mediation analysis, so that the components shown to be effective could be used to target future health promotion and wellness strategies (31–33).

Healthy Team Healthy U was implemented at Oregon Health & Science University (OHSU) as a wellness offering for benefited employees during 2011–2014. The aim of this study was to discover whether our team-based intervention paradigm, when combined with a scalable digital platform could be effectively translated to a worksite setting among a wide spectrum of hospital, clinic, and university workers.

## Materials and Methods

### Study design

Data were collected using a prospective cohort study to evaluate feasibility, health behaviors, outcomes, and participant perceptions of the HTHU intervention’s initial 12-session program (HTHU Level-1) and the 12-session booster (HTHU Level-2), the following year. The pre- and post-intervention and assessments included questionnaires and biometric indices of blood pressure, height, and body weight. The interventions were among benefited employees at the OHSU. The initial cohort was engaged in Level-1 during work year 2011–2012. The second cohort started the Level-1 intervention during 2012–2013. Those completing the Level-1 intervention could participate in the Level-2 health promotion and wellness intervention the subsequent year. A longitudinal analysis consisted of those participants completing all four questionnaires and/or biometric assessments. This latter analysis was used to assess the durability of the Level-1 intervention and potential enhancement effects of the Level-2 program. Objective targets included reductions in weight and BMI for those overweight and obese and reduction in blood pressure among employees with pre-hypertension and hypertension.

The study followed the recommendations of the STROBE (41) statement for observational studies. Employees provided informed consent at enrollment for confidential data collection, and those consenting were offered participation in pre and post survey collection and biometric measures. There was no incentive either for survey completion or biometric assessments. OHSU’s Institutional Review Board approved the study and its procedures (IRB number #6638).

### Setting and participants

All benefited OHSU employees (*N* ~ 8,500–9,500, depending on the year) were eligible to participate in HTHU as one of the university’s healthy options. OHSU has a central campus and hospital with additional suburban and rural campuses and clinics. With numerous departments, centers, and support services, OHSU employee education levels range from less than a high school diploma to multiple doctorate degrees.

Recruitment to the program included web-based information, health fairs, and informational sessions. All participants were encouraged to have blood pressure, height, and weight measured pre and post intervention and to complete an anonymous self-report questionnaire before and after the Level-1 and Level-2 interventions. Email and phone communications, posters, and in-person communication encouraged participation and assessments.

### Measures

Study staff, either by walk-in or appointment, conducted all biometric assessments. Weight was measured using a digital electronic scale. Participants were weighed without shoes and wore indoor clothing. Height was measured using a stadiometer. Height and weight were used to calculate BMI in kilogram per square millimeter. Blood pressure measurement was performed after a 5-min seated rest with an automated aneroid sphygmomanometer, with 2 min between subsequent measurements. An average of three measurements was made for both systolic and diastolic blood pressure.

Employees volunteered to complete a brief personal health behavior assessment, written at an approximately sixth grade level. The surveys were derived from validated instruments used in prior studies (25–37). During 2011–2012 and 2012–2013, employees participating in HTHU Level-1 completed a 28-item survey prior to participation and a 39-item survey after the program. During 2012–2013 and 2013–2014, employees participating in HTHU Level-2 completed a 25-item survey prior to participation and a 39-item survey after the program’s conclusion. Each survey contained questions that included an estimation of daily servings of fruits and vegetable intake, daily PA of ≥30 min, and the intensity of exercise. The perceived level of employee wellbeing, stress, happiness, and depression were each reported on a 7-point Likert agreement scale. Seven-point and other linear scales have been found to have appropriate for assessment of mood and wellbeing as a screening device, and found to be useful clinically (42–46). Post-surveys included additional queries regarding the benefits of specific elements of the intervention and overall program satisfaction, including expectations, overall evaluation, and agreement with the perceived benefits of team sessions, goals, and online activities.

### Intervention

Healthy Team Healthy U™ is a series of 12-session health promotion programs with sessions focusing on specific healthy lifestyle behavior changes through a sequence team-based learning modules. The program integrates health behaviors and activities that target healthy nutrition, PA, safety, stress reduction, mood improvement, and sleep quality and quantity, combined with skill acquisition, goal setting, and team member support and feedback. Each session was structured with the following framework: (1) a team meeting addressing specified health knowledge, behavioral skills, and norms, (2) goal setting with (3) monitoring and feedback. All sessions have learning objectives. The scope and sequence of the learning activities were designed to build on previous meetings for each topic or introduce a new health topic.

A booster 12-session curriculum (Level-2) was added to further enhance health behaviors. Level-2 had unique content that built upon the initial intervention and was designed to sustain engagement and enhance health behaviors and strategies. Employees participating in the Level-1 program were eligible to engage in Level-2 the following academic year.

Our prior team-based intervention paradigm was combined with a digital platform with advanced activity tracking and monitoring and social networking functionality. Online assets included exercise and cooking videos created specifically for the interventions, educational gamified activities, and dedicated social feeds. A point system assisted accountability, motivation, and peer support. All session materials could be accessed on the platform using a computer, electronic tablet, or smartphone.

Level-1 participants received a digital pedometer as an activity tracker. Participants in Level-2 received or had access to resistance bands for strength training.

### Statistical analysis

Three datasets are used to assess effects of the intervention: (1) the pre- and post-surveys and/or biometrics (BMI and blood pressure) for the initial (Level-1) program; (2) the pre- and post-surveys and/or biometrics for the booster (Level-2); and (3) the longitudinal analysis of those employees who completed all four questionnaires or biometric assessments. The longitudinal analysis assists in understanding the durability of the initial intervention, e.g., whether employees reverted to their prior lifestyle behaviors and/or biometrics during the 1-year span between participation in the initial and booster intervention. The analysis also helps assess whether the booster further improved health behaviors and outcomes produced by the initial intervention.

Results for each dataset were analyzed with repeated measure ANOVAs. The main intervention and the booster were each provided during consecutive years. The data from both years were combined for analysis. The analysis includes the determination of effect size, used to help determine the magnitude of the program’s impact on outcomes, without respect to sample size (47, 48). Calculation of effect size is especially useful when the measurements are in a Likert style agreement scale, as used in our pre- and post-assessments (48).

To assist in assessing whether there was a selection bias among employees volunteering for post intervention measurements, we compared employees who completed the initial assessments only to those employees who completed both pre and follow-up measurements. To assess whether the longitudinal population was an accurate representation of the participant population, we compared the longitudinal sample with those who completed the pre- and post- of the initial intervention.

## Results

### Participants and data collection

During the 2011–2012 and 2012–2013 academic years, 3,780 employees participated in Level-1. Overall, 2,817 (74%) of the total number of participants completed the voluntary pre-intervention questionnaire, and of those 986 (35%) completed post-surveys. During this period, 829 employees (22% of the total) had a pre-intervention biometric assessment, and of those, and 473 participants completed pre and post BMI assessments and 466 participants had pre and post blood pressure determinations. Table 1 shows the number of employees completing the surveys and biometrics of the Level-1 program during the first 2 years.

**Table 1:** **Number of participants in the initial (HTHU 1.0) intervention**.

	2011–2012	2012–2013	Total
Completed pre surveys only	2,270	547	2,817
Completed pre- and post-surveys	860	126	986
Completed pre biometric	565	264	829
Completed pre and post BMI	335	138	473
Completed pre and post BP	331	135	466

As shown in Table 2, among Level-2 participants, 867 employees (46% of the total) completed the voluntary pre-intervention health survey, with 212 of those completing both pre- and post-surveys. During this period, 470 Level-2 participants (25% of the total) had a pre-intervention biometric measurement, and of those, 214 employees had both pre- and post-weight measurements and 213 had both pre and post blood pressure assessments.

**Table 2:** **Number of participants in booster (HTHU 2.0) intervention**.

	2012–2013	2013–2014	Total
Completed any assessment	788	286	1,074
Completed pre surveys only	601	266	867
Completed pre- and post-surveys	139	73	212
Completed pre biometric	391	79	470
Completed pre and post BMI	181	33	214
Completed pre and post BP	181	32	213

The longitudinal sample consisted of those participants identified as completing either all four surveys or all four biometric assessments. Among those in the longitudinal analysis, 68 participants completed all 4 height and weight assessments, 71 completed all blood pressure measurements, and 88 participants completed all 4 questionnaires.

### Measures

Pre-intervention baseline characteristics of employees engaged in HTHU are provided in Table 3. It includes all participants who completed only the initial surveys and/or biometrics, as compared to participants completing pre- and post-surveys and/or biometrics. Among those two participant groups, there were no differences for mean age, weight, BMI, systolic and diastolic blood pressure measures, self-report of fruit and vegetable intake, or daily PA. There was a statistically significant difference in perceived stress (*p* = 0.02) among those who completed only the initial measurements, although the mean difference was just 0.16 on a 7-point scale of agreement.

**Table 3:** **Comparison among those who completed only pre-test and those who completed both pre- and post-tests in HTHU 1.0 program (mean **±** SD)**.

Variable	Pre-test only	Both pre- and post-tests	*p*-Value (unpaired)
Age	42.0 ± 11.5	41.9 ± 11.5	0.93
Fruits and vegetables servings/day	3.57 ± 1.52	3.62 ± 1.52	0.44
Days/week of physical activity for at least 30 min	3.98 ± 1.76	3.84 ± 1.78	0.06
Stress at work	4.68 ± 1.64	4.52 ± 1.68	0.02
Happiness	5.33 ± 1.25	5.41 ± 1.22	0.11
Depression	2.61 ± 1.51	2.54 ± 1.45	0.31
Systolic blood pressure	118.6 ± 13.9	118.9 ± 15.1	0.7
Diastolic blood pressure	76.4 ± 10.5	75.1 ± 10.2	0.07
Weight	173.04 ± 43.6	168.6 ± 41.6	0.14
BMI	27.9 ± 6.83	27.1 ± 5.84	0.06

Among participants assessed for Level-2, no statistically significant differences were found between those who completed only the pre-assessment surveys and/or biometrics prior to the booster intervention and participants completing both the pre- and post-surveys and biometric assessments (Table 4). The employee samples were similar with respect to age, mean systolic and diastolic blood pressure, weight and BMI, self-reported daily intake of fruits and vegetables, and number of days/week of ≥30 min PA, stress, depression, and happiness.

**Table 4:** **Comparisons of employees with pre-test only and employees with both pre- and post-tests in the booster (HTHU 2.0) program (mean **±** SD)**.

Variable	Only pre-tests	Both pre- and post-tests	*p*-Value (unpaired)
Age	47.9 ± 10.7	47.5 ± 15.1	0.93
Fruit/vegetable servings/day	4.5 ± 1.25	4.62 ± 1.22	0.23
Days/week of physical activity ≥30 min	4.63 ± 1.62	4.57 ± 1.52	0.6
Stress at work	4.44 ± 1.65	4.21 ± 1.68	0.076
Happiness	5.03 ± 1.39	5.06 ± 1.44	0.78
Depression	2.5 ± 1.46	2.48 ± 1.54	0.85
Weight	169.6 ± 44.9	174.1 ± 43.1	0.26
Systolic blood pressure	117.1 ± 13.9	117.0 ± 14.1	0.94
Diastolic blood pressure	75.6 ± 10.2	74.8 ± 9.87	0.38
BMI	27.6 ± 6.64	28.0 ± 6.44	0.47

Among those in the longitudinal analysis, no differences were found for age, BMI, blood pressure, daily PA, or daily fruit and vegetable intake when compared to participants prior to the initial (Level-1) intervention who completed both pre and post measurements.

### Program outcomes: Initial wellness intervention

Table 5 shows outcome assessments that include both the proximal (health behaviors of diet and PA) the distal outcomes of biometrics and self-report of mood, stress levels, and healthy behaviors before and after the initial intervention (Level-1). Health outcomes for participants at the conclusion of the initial intervention include significant reductions in mean systolic and diastolic blood pressure among participants, with greatest decrements among employees with pre-hypertension (−4.7/−2.7) and hypertension (−13.1/−5.6) (all *p* < 0.0001), significant reductions in body weight and BMI for all employees assessed (*p* < 0.0001), with greatest change among those initially in the overweight (BMI = 25–29.9) (*p* < 0.0001) and obese (BMI ≥30) (*p* < 0.002) categories.

**Table 5:** **Initial year (1.0) wellness program outcomes**.

	*F* value	*p*-Value (paired)	Partial **η**^2^	Obs	Mean diff	Mean Pre	Std pre	Mean post	Std post
Change in SBP	23.93	<0.0001	0.05	466	−2.43	118.96	15.13	116.53	14.08
Change in DBP	19.04	<0.0001	0.04	466	−1.6	75.15	10.24	73.56	10.09
Change in SBP – hypertensive	43.41	<0.0001	0.51	43	−13.07	149.19	7.8	136.12	13.35
Change in SBP – pre-hypertensive	34.81	<0.0001	0.17	171	−4.7	127.83	5.61	123.13	11.33
Change in DBP – hypertensive	16.62	<0.001	0.28	43	−5.58	89.33	8.35	83.74	10.8
Change in DBP – pre-hypertensive	17.57	<0.0001	0.09	171	−2.67	79.91	7.9	77.23	8.96
Change in BMI	61.73	<0.0001	0.12	439	−0.31	27.11	5.84	26.8	5.88
Change in BMI – obese	10.99	<0.01	0.09	108	−0.3	35.43	4.74	35.13	4.95
Change in BMI – overweight	29.1	<0.0001	0.17	139	−0.44	27.08	1.41	26.63	1.64
Change in weight	77.15	<0.0001	0.15	448	−2.02	168.72	41.35	166.7	41.27
Change in weight – obese	17.57	<0.0001	0.14	108	−2.25	220.36	36.56	218.11	36.68
Change in weight – overweight	33.42	<0.0001	0.19	141	−2.59	171.59	22.54	169	23.46
Change in stress	0.23	0.63	0	935	−0.02	4.52	1.69	4.5	1.7
Change in stress – stressed	93.59	<0.0001	0.24	298	−0.58	6.33	0.47	5.74	1.07
Change in happiness	36.42	<0.0001	0.04	935	0.22	5.41	1.22	5.63	1.09
Change in happiness – unhappy	39.61	<0.0001	0.57	31	2.03	1.84	0.37	3.87	1.65
Change in depression	31.56	<0.0001	0.03	933	−0.26	2.55	1.45	2.29	1.34
Change in depression – depressed	59.14	<0.0001	0.65	33	−2.48	6.06	0.24	3.58	1.84
Change in FV consumption	406.14	<0.0001	0.3	939	0.77	3.63	1.52	4.4	1.29
Change in FV consumption <5	584.51	<0.0001	0.41	836	0.94	3.28	1.21	4.22	1.2
Change in days of 30 min exercise	177.16	<0.0001	0.16	909	0.68	3.95	1.68	4.63	1.48
Change in days of 30 min exercise <4	466.52	<0.0001	0.54	392	1.59	2.36	0.73	3.95	1.39

*SBP, systolic blood pressure; DBP, diastolic blood pressure; BMI, body mass index; FV, fruits and vegetables*.

Other favorable self-reported distal outcomes include an increase in perceived happiness among all participants, including an increase among those initially reporting unhappiness (both *p* < 0.0001), and reduction in overall feelings of depression among employees, with greatest improvement among those initially reporting feeling depressed (both *p* < 0.0001). Stress reduction was found among those with higher self-reported work stress (*p* < 0.0001). Although a small, yet statistically significant increase in stress occurred among those with initial low stress after the program (*p* < 0.0001), these lower stressed employees remained within the low stress category after the intervention. Employees indicated improved happiness after the intervention, including those who self-reported unhappiness prior to the program (both *p* < 0.0001).

Self-report of daily intake of fruits and vegetables increased 22% (*p* < 0.0001), despite a slight regression to the mean among those already eating five or more servings each day. Daily PA increased 18.7% (*p* < 0.0001), which was driven by the increase among those who started with <4 days of PA each week.

Table 6 shows survey results regarding perception of health, days of moderately vigorous and vigorous PA, strength training, self-efficacy, perception of peer influence, engagement in health behaviors, and assessments of wellbeing before and after the initial intervention. Improvements include knowledge of how to balance diet and exercise and confidence in ability to strength train (both *p* < 0.0001). Employees reported enhanced overall health, including health within the past 3 months (both *p* < 0.0001). Average days/week of strength training increased 48%, while moderately intense PA increased 25% and intense exercise increased 24% (all *p* < 0.0001). In addition, employees reported being encouraged to be physically active by co-workers (*p* < 0.0068) and exercised more with fellow employees (*p* < 0.0001).

**Table 6:** **Health, diet, exercise, and co-worker influence before and after initial (HTHU 1.0) program**.

	*N*	Pre-mean	Post-mean	Mean diff	SE	*t*	*p*-Value (paired)
General health	965	3.39	3.64	−0.24	0.02	−11.74	<0.0001
Past 3 months health	944	3.31	3.61	−0.30	0.02	−13.02	<0.0001
Recommended exercise	958	4.04	4.39	−0.35	0.04	−7.84	<0.0001
Recommended water	954	5.54	5.81	−0.27	0.06	−4.62	<0.0001
Recommended fruits/vegetables	954	4.46	4.95	−0.49	0.05	−10.47	<0.0001
Balance PA and diet for weight^a^	961	5.41	6.12	−0.71	0.04	−17.29	<0.0001
Days/week intense activity	934	2.15	2.66	−0.50	0.05	−9.24	<0.0001
Days/week moderate activity	933	2.95	3.69	−0.74	0.07	−11.34	<0.0001
Days/week strength training	933	1.57	2.33	−0.76	0.06	−13.72	<0.0001
Days/week any activity	934	3.95	4.70	−0.75	0.07	−10.87	<0.0001
Co-workers encourage eating	124	4.31	4.60	−0.29	0.15	−1.90	0.0603
Co-workers encourage activity	125	4.40	4.79	−0.39	0.14	−2.76	0.0068
Purchase healthy food at work	913	4.94	5.12	−0.18	0.04	−4.25	<0.0001
Exercise with co-workers	125	2.01	2.58	−0.57	0.12	−4.68	<0.0001
Strength training self-efficacy	126	4.96	5.63	−0.67	0.13	−5.38	<0.0001

*^a^Ability to balance physical activity and diet to lose weight or stay at a healthy weight*.

### Booster program and outcomes

Table 7 shows proximal outcomes of health behaviors, and distal outcomes of biometrics and self-report of mood and stress, before and after the booster intervention (Level-2). Among employees with pre-hypertension, there was a 5.3 mmHg reduction in systolic blood pressure (*p* < 0.0001) and a 2.9 mmHg lowering of diastolic blood pressure (*p* < 0.05). Among employees with hypertension, systolic blood pressure decreased 9.0 mmHg (*p* < 0.01) and a 3.3 mmHg reduction in diastolic blood pressure (*p* < 0.05). Weight was significantly reduced among the overweight (*p* < 0.05) and obese (*p* < 0.01) employees, with a significant BMI reduction in BMI observed among obese participants (*p* < 0.01).

**Table 7:** **Booster year (2.0) outcome results**.

	*F* value	*p*-Value (paired)	Partial **η**^2^	Obs	Mean diff	Mean pre	Std pre	Mean post	Std post
Change in SBP	8.27	<0.01	0.04	213	−1.86	117.04	14.15	115.17	13.61
Change in DBP	7.99	<0.01	0.04	213	−1.32	74.8	9.88	73.47	9.71
Change in SBP – hypertensive	10.91	<0.01	0.39	18	−9	147.67	5.37	138.67	12.65
Change in SBP – pre-hypertensive	20.99	<0.0001	0.26	60	−5.27	127.65	5.34	122.38	10.36
Change in DBP – hypertensive	6.45	<0.05	0.28	18	−3.28	89.33	8.25	86.06	8.07
Change in DBP – pre-hypertensive	10.38	<0.05	0.15	60	−2.92	80.22	7.98	77.3	8.73
Change in BMI	16.56	<0.0001	0.07	214	−0.26	28.02	6.45	27.76	6.37
Change in BMI – obese	10.71	<0.01	0.14	68	−0.53	35.58	5.11	35.05	5.34
Change in BMI – overweight	4.83	0.12	0.07	63	−0.2	27.42	1.47	27.22	1.7
Change in weight	18.1	<0.0001	0.12	139	−1.68	174.16	43.15	172.48	42.35
Change in weight – obese	11.16	<0.01	0.14	68	−3.32	218.84	35.59	215.53	36.25
Change in weight – overweight	5.64	<0.05	0.08	63	−1.42	175.8	22.23	174.37	21.87
Change in stress	1.15	0.29	0.01	209	0.13	4.21	1.69	4.34	1.67
Change in stress – stressed	38.74	<0.0001	0.45	48	−1.31	6.46	0.5	5.15	1.43
Change in happiness	23.08	<0.0001	0.1	208	0.47	5.07	1.44	5.53	1.34
Change in happiness – unhappy	7.37	<0.05	0.42	11	1.27	1.82	0.4	3.09	1.51
Change in depression	0.36	0.55	0	207	−0.07	2.48	1.54	2.42	1.52
Change in depression – depressed	10.47	<0.01	0.49	12	−2.33	6.33	0.49	4	2.26
Change in FV servings	76.19	<0.0001	0.27	208	0.63	4.63	1.22	5.26	1.23
Change in FV servings <5	98.37	<0.0001	0.39	15	0.8	4.14	0.84	4.94	1.16
Change in ≥30 min exercise	72.49	<0.0001	0.26	209	0.84	4.57	1.52	5.41	1.4
Change in ≥30 min exercise <4	44.9	<0.0001	0.54	40	1.7	2.68	0.47	4.38	1.37
Changes in sleep hours – ≤6 h	17.44	<0.001	0.47	21	0.71	1.81	0.4	2.52	0.75
Changes in sleep quality	18.88	<0.0001	0.08	209	0.51	4.22	1.74	4.73	1.56

*SBP, systolic blood pressure; DBP, diastolic blood pressure; BMI, body mass index; FV, fruits and vegetables*.

Self-report surveys revealed positive distal outcomes, including a reduction in feelings of stress among those who indicated that they had worksite stress prior to the booster program (*p* < 0.0001). Similar to initial intervention findings, those with lower stress scores had a mean increase in stressful feelings after the intervention (*p* < 0.0001). However, the low-stressed employee change within the 1–7 Likert agreement scale was only 0.6, and the resultant mean change remained within the low-stressed level. Improvement in perceived happiness occurred among all employees (*p* < 0.0001), including those initially unhappy prior to the intervention (*p* < 0.02). The index of self-reported depression was reduced overall (*p* < 0.0001) and among those reporting initial depression (*p* < 0.01). Dietary self-reports revealed a significant increase in daily servings of vegetables and fruits (*p* < 0.0001), with an average intake exceeding the minimum of five servings/day the program recommended. There was also a significant increase in daily PA of ≥30 min or more (*p* < 0.0001). Because the booster program added a sleep intervention, we assessed self-report of sleep outcomes. Sleep quality improved, overall (*p* < 0.0001) and although sleep did not increase among all respondents, those reporting <6 h of sleep prior to the booster significantly increased sleep duration (*p* < 0.001).

Table 8 reveals survey results regarding self-efficacy, perception of peer influence, health behaviors, and wellbeing before and after the HTHU booster program. Participants reported improved perception of their overall health and specifically, their health during the past 3 months (both *p* < 0.0001). Days/week of being engaged in strength training increased 21.8% (*p* < 0.0001) after the booster. PA increased, with a 17.2% increase in moderately intense and 21.2% rise in intense PA (all *p* < 0.0001). Along with these improvements, participants had greater agreement that co-workers encouraged them to be more active and have healthier eating habits (both *p* < 0.0001) and employees were more engaged in PA with co-workers (*p* < 0.0001).

**Table 8:** **Health, diet, exercise and co-worker influence before and after booster (HTHU 2.0) program**.

	*N*	Pre-mean	Post-mean	Mean diff	SE	t	*p*-Value (paired)
General health	214	3.45	3.68	−0.23	0.05	−4.89	<0.0001
Past 3 months health	214	3.39	3.60	−0.21	0.05	−3.98	<0.0001
Recommended exercise	210	4.96	5.24	−0.29	0.07	−4.06	<0.0001
Recommended water	208	6.38	6.59	−0.21	0.07	−2.99	0.0031
Recommended fruits/vegetables	211	5.58	5.89	−0.31	0.07	−4.46	<0.0001
Balance PA and diet for weight^a^	210	5.45	6.08	−0.63	0.10	−6.64	<0.0001
Days/week intense activity	212	2.92	3.54	−0.62	0.11	−5.59	<0.0001
Days/week moderate activity	210	3.60	4.22	−0.62	0.12	−5.14	<0.0001
Days/week strength training	212	2.66	3.34	−0.68	0.12	−5.8	<0.0001
Days/week any activity	210	4.82	5.44	−0.62	0.12	−5.22	<0.0001
Co-workers encourage eating	209	4.30	5.13	−0.83	0.10	−8.32	<0.0001
Co-workers encourage activity	210	4.20	5.07	−0.87	0.10	−8.71	<0.0001
Purchase healthy food at work	212	5.10	5.47	−0.37	0.09	−4.14	<0.0001
Exercise with co-workers	210	2.44	2.92	−0.48	0.11	−4.44	<0.0001
Strength training self-efficacy	211	5.09	5.59	−0.50	0.10	−4.97	<0.0001

*^a^Ability to balance physical activity (PA) and diet to lose weight or stay at a healthy weight*.

### Longitudinal analysis

**Figure 1:**
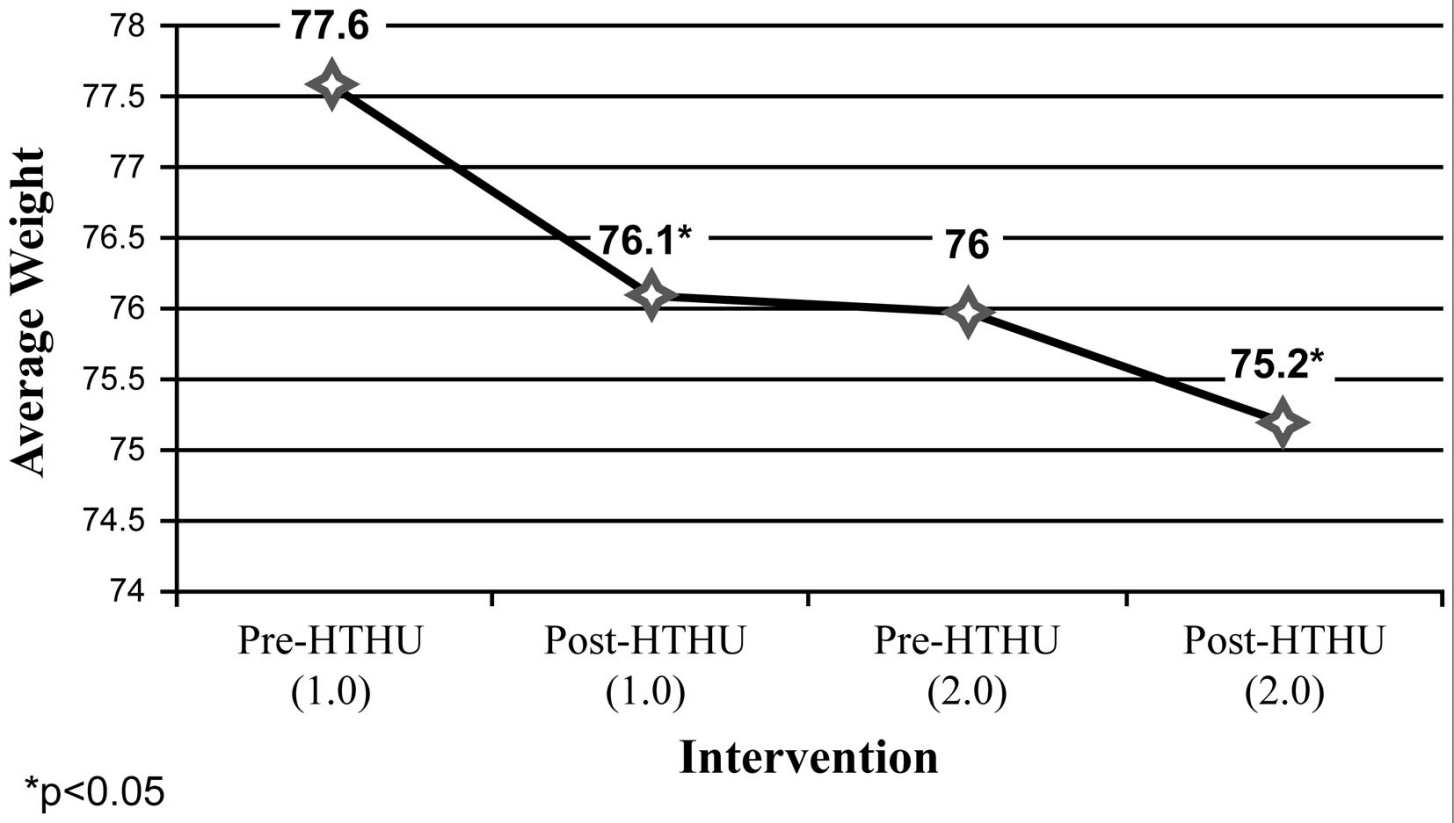
**Weight loss (kilogram) (all employees)**.

**Figure 2:**
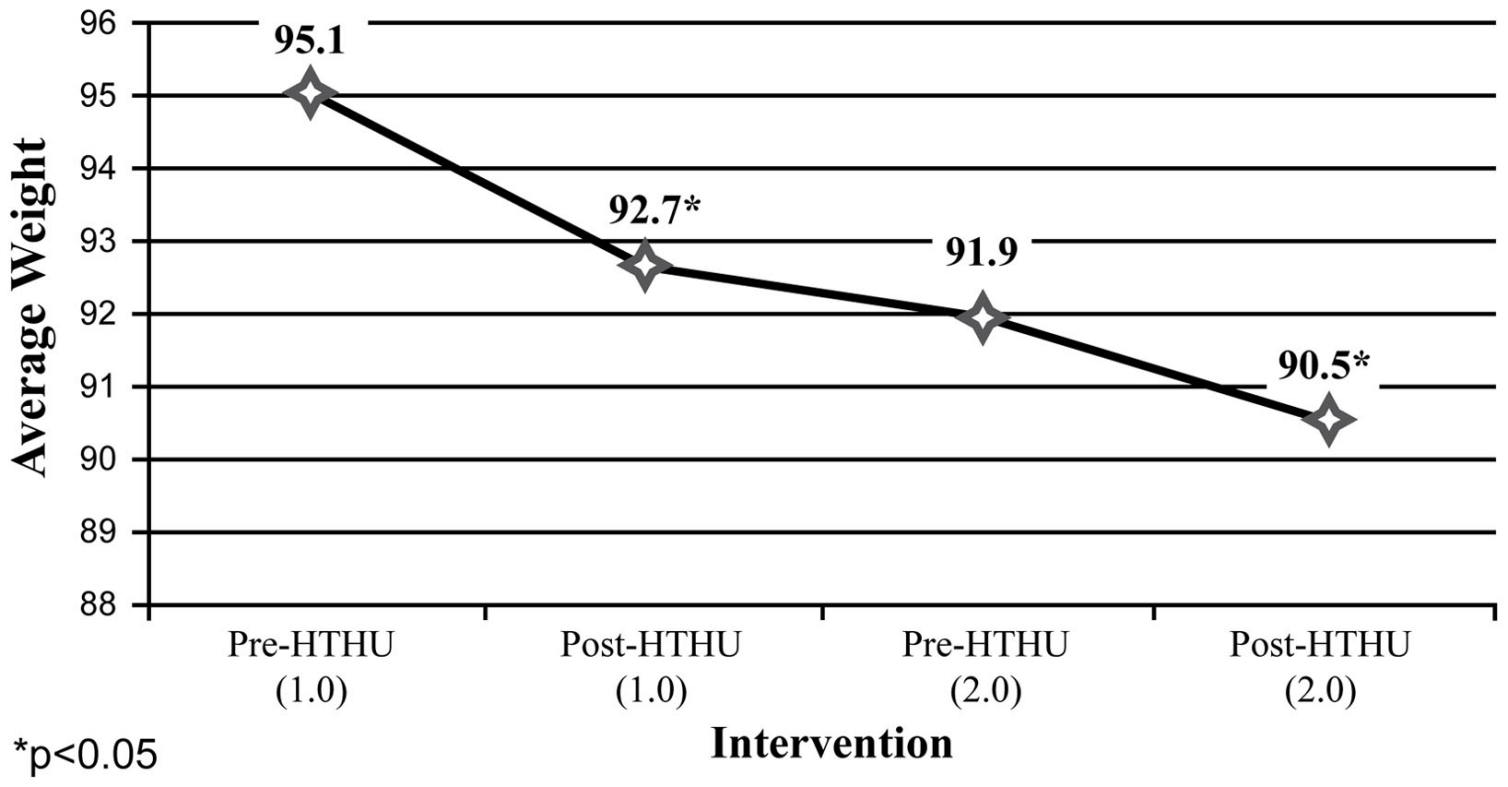
**Weight loss (kilogram) (employees **≥**30 BMI)**.

**Figure 3:**
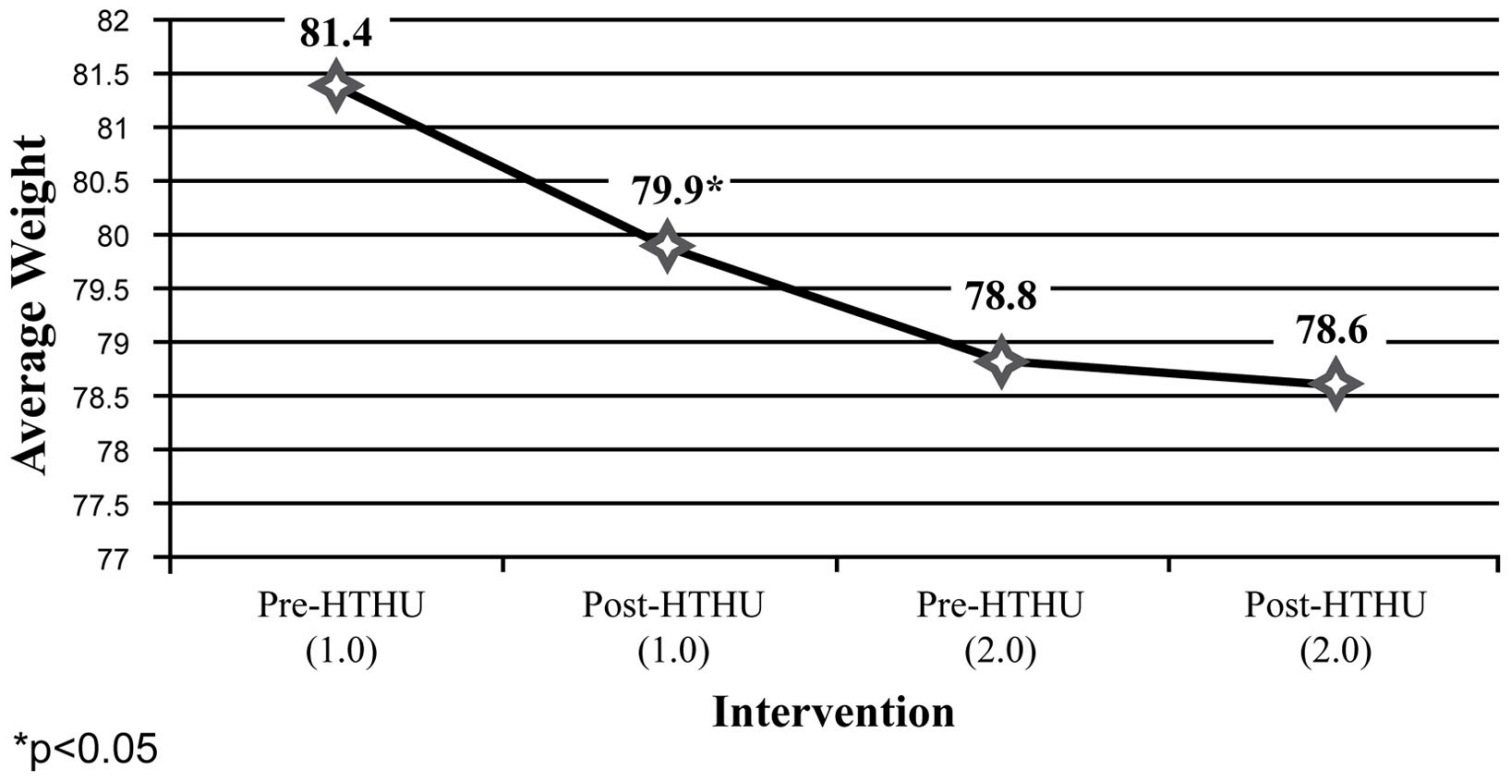
**Weight loss (kilogram) (employees 25 **<** 30 BMI)**.

Weight reduction was found among all employees (Figure 1), with sustained loss during the time period after completion of the initial Level-1 intervention and prior to participating in the booster the following academic year. Further weight loss was achieved after the booster, with a mean total decrease after both interventions of 2.36 kg. Greater weight loss and a similar pattern of sustained loss between interventions were found among the obese and overweight employees (*N* = 40). Among employees who were initially obese (*N* = 22), there was an overall 4.58 kg reduction in weight (Figure 2). The weight reductions after the initial intervention persisted, without returning to baseline weight between the interventions, with further weight reductions following the booster. Likewise, Figure 3 shows the weight change among employees in the overweight category (*N* = 18). Those initially overweight had weight loss that persisted between interventions with a further reduction in weight at the conclusion of the booster. There was insufficient data to assess blood pressure among the hypertensive and pre-hypertensive subjects among this group.

**Figure 4:**
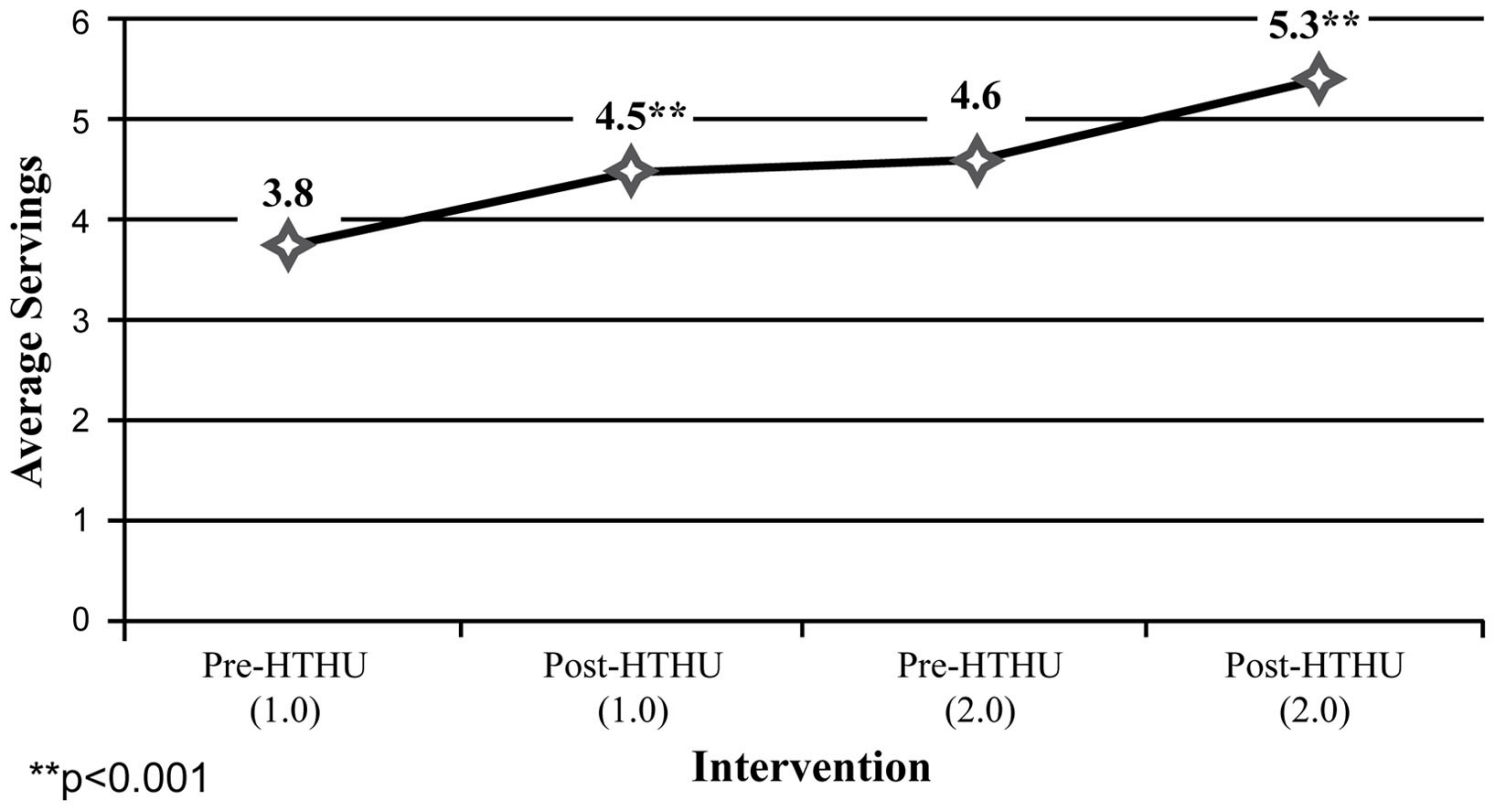
**Fruits and vegetables servings (all employees)**.

**Figure 5:**
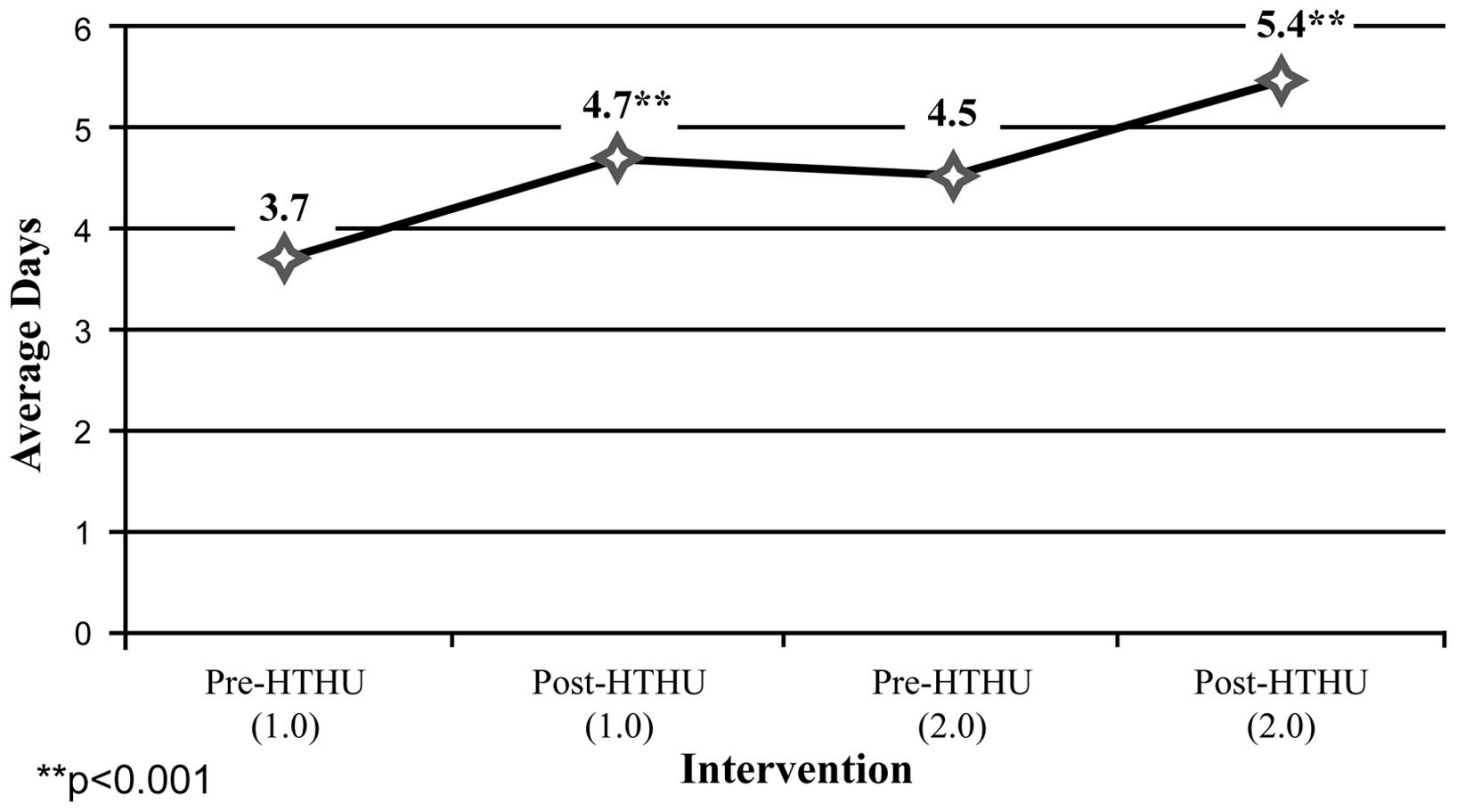
**Days/week of **≥**30 min physical activity**.

Among the self-report measures, participants indicated a 21% increase of daily fruit and vegetable intake after Level-1, which was sustained leading up to the booster intervention (Figure 4). After the booster, participants reported an additional 19.7% average daily increase in fruit and vegetable intake. Daily PA of 30 min among participants increased 27% after the initial intervention. This increase was sustained during the intervening year, and rose 14.95% to an average 5.4 days of PA/week (Figure 5). In addition, a reduction of perceived stress level among employees was observed after the initial wellness program, declining further during the year, prior to the booster intervention. There were insufficient numbers of employees with depression who completed all surveys for analysis.

### Employee satisfaction

Employee assessments of HTHU Level-1 and Level-2 are shown in Tables 9 and 10, respectively. The responses were measured by employees indicating level of agreement to a statement about program components using a scale from 1 (strongly disagree) to 7 (strongly agree). The overall assessment of the program was made using a 1 (not helpful) to 5 (very helpful) scale of agreement. Among all participants, 98% reported they would recommend HTHU to their co-workers. The highest regarded elements of the programs were nearly identical after Level-1 and Level-2 and included the team sessions, online activities, use of the pedometer and the multi-week step challenge, goal setting, and use of the Wellness Guide, a multi-chapter resource providing more in-depth information of each health topic. No program feature had a neutral or negative rating.

**Table 9:** **Employee evaluation of initial intervention (HTHU 1.0) program**.

Measure	*N*	Mean	SD	Range
Team sessions valuable	130	5.49	1.18	1–7, strongly disagree–strongly agree
Wellness guide valuable	131	5.31	1.21	1–7, strongly disagree–strongly agree
Pedometer valuable	130	6.15	1.05	1–7, strongly disagree–strongly agree
Online activities valuable	130	5.39	1.18	1–7, strongly disagree–strongly agree
Goals valuable	129	5.74	0.96	1–7, strongly disagree–strongly agree
Cooking videos/recipes valuable	128	4.45	1.44	1–7, strongly disagree–strongly agree
Strength training challenge	129	4.84	1.49	1–7, strongly disagree–strongly agree
Pedometer challenge valuable	128	6.09	0.88	1–7, strongly disagree–strongly agree
Strength training videos	129	4.57	1.39	1–7, strongly disagree–strongly agree
Strength train regularly	130	4.44	1.66	1–7, strongly disagree–strongly agree
Make healthier food choices	128	5.33	1.35	1–7, strongly disagree–strongly agree
Make healthier beverage choices	130	5.10	1.53	1–7, strongly disagree–strongly agree
Expectations met	130	5.85	1.04	1–7, strongly disagree–strongly agree
Would recommend HTHU	131	6.21	0.96	1–7, strongly disagree–strongly agree
Incentive was a motivator	126	6.23	1.14	1–7, strongly disagree–strongly agree
Overall HTHU evaluation	131	4.19	0.81	1–5, not helpful–helpful

**Table 10:** **Employee Evaluation of booster (HTHU 2.0) program**.

Measure	*N*	Mean	SD	Range
Team sessions valuable	181	5.44	1.18	1–7, strongly disagree–strongly agree
Wellness guide valuable	182	5.21	1.25	1–7, strongly disagree–strongly agree
Pedometer valuable	180	6.14	1.05	1–7, strongly disagree–strongly agree
Online activities valuable	180	5.35	1.15	1–7, strongly disagree–strongly agree
Goals valuable	179	5.65	0.96	1–7, strongly disagree–strongly agree
Cooking videos valuable	131	4.40	1.45	1–7, strongly disagree–strongly agree
Strength training challenge	180	4.88	1.40	1–7, strongly disagree–strongly agree
Pedometer challenge valuable	179	6.07	0.96	1–7, strongly disagree–strongly agree
Strength training videos	141	4.60	1.38	1–7, strongly disagree–strongly agree
More physically active	49	5.08	1.30	1–7, strongly disagree–strongly agree
Strength train regularly	180	4.36	1.60	1–7, strongly disagree–strongly agree
Make healthier food choices	177	5.24	1.39	1–7, strongly disagree–strongly agree
Healthier beverage choices	181	5.08	1.52	1–7, strongly disagree–strongly agree
Expectations met	181	5.80	1.02	1–7, strongly disagree–strongly agree
Would recommend HTHU	182	5.83	1.07	1–7, strongly disagree–strongly agree
Incentive was a motivator	129	6.21	1.19	1–7, strongly disagree–strongly agree
Overall HTHU evaluation	182	4.14	0.82	1–5, not helpful–very helpful

## Discussion

Healthy Team Healthy U is the translation to a diverse workplace setting of health promotion interventions previously assessed in randomized clinical trials among distinct populations including athletes, fire fighters, law enforcement employees, and other groups (25–37). The results of this prospective cohort study support the transferability and sustained positive impact on health behaviors and outcomes of a research tested, team-based intervention paradigm combined with a digital platform. The intervention improved biometric indices of blood pressure, body weight, BMI, diet, PA frequency and intensity, fruit and vegetable intake, mood, stress levels, and sleep quality and quantity among a diverse employee population. Employee participants had similarities with the biometrics and self-report health behaviors of typical of U.S. employees with regard to BMI, blood pressure, daily PA, and fruit and vegetable intake (49–52).

The theoretical underpinnings highlight how the program may create health behavior change. The team sessions, knowledge acquisition, review, and feedback of weekly health goals and web-based social communication capabilities among team members between each session, provided employees with direct and vicarious modeling featured in the Theory of Social Learning (40). These activities foster healthy normative beliefs and behaviors. The Theory of Reasoned Action, which postulates that attitudes influence intentions, which are theorized precursor of behaviors, were paramount in structuring the curriculum and web-based intervention (38). Sessions imparted how health behaviors influenced each employee’s physical and emotional health. The team-based intervention used in this and our prior research-tested model appears to have reflected improved employee cohesion with task oriented social support, as employees reported their co-workers encouraged them to be more active, have healthier eating habits and employees had greater engagement in PA together (27, 29).

Goals were designed to practice healthy behaviors that could mitigate unhealthy risks, creating a positive attitude, and intention toward the particular health behavior. Although clinically significant health outcomes are the desired effect, this can take weeks or months to occur. Real time activity tracking of personal and team progress delivered immediate feedback to the participant, which could influence short-term positive feedback. This tangible, instantaneous outcome may have generated the social reward underlying both theories of Social Learning and Reasoned Action.

In addition to the health benefits of health promotion strategies that focus on PA and diet, wellness strategies concentrating on mood, stress, and sleep may be critical to reducing medical costs and improving work productivity (15–17, 53–55). Workplace stress is common, costly, and highly related to presenteeism, lost workdays, and a higher use of health care services (15, 53). Among employees with depression, there are numerous indirect costs and loss of productivity, with more than one of every three with depression developing a short-term disability during a given year (54). Reduced sleep duration, especially <6 h within a 24-h period, is related to obesity, metabolic syndrome, and type 2 diabetes (55–58). We found reductions in self-reported depression, lower stress scores among those indicating heightened stress prior to the intervention, and improvement in happiness. We also found improved quality of sleep overall, and increased sleep duration among those with lower sleep hours (≤6) after the booster intervention.

Both the initial and booster intervention significantly reduced body weight, with greatest losses among obese and overweight employees. Obese participants had a 4.8% reduction in body weight assessed with the longitudinal analysis at 1 year follow-up. Among employees in the obese category, the 4.58-kg mean body weight reduction was similar or better than the weight loss reported with popular commercial weight loss and research-based programs and those using coaching or counseling, despite HTHU not being a specific weight reduction intervention (59–62).

The positive blood pressure and weight loss outcomes were consistent with the self-reported improvements in diet and PA. After the initial intervention, employees’ fruit and vegetable intake increased 22%, and increased an additional 14% after the booster, exceeding the recommended average of a minimum of five servings/day (51). Importantly, this threshold recommended by the Centers for Disease Control may be an optimal level for cardiovascular and cancer prevention and reduction in all-cause mortality (18, 63). Likewise, after the initial and booster program, average daily PA increased to 5.4 days, while strength training increased beyond the United States Department of Health and Human Services recommendations (64).

Among the longitudinal employee sample, both objective and self-reported positive outcomes were sustained during the time between the initial intervention and the booster intervention the following year. Specifically, average weight loss occurring after the initial intervention was maintained among all employees measured, including those in the obese and overweight categories in the time span leading up to the booster intervention the subsequent year. Likewise, increases in daily fruit and vegetable intake and daily PA reported after the initial intervention persisted between the end of Level-1 and the beginning of the Level-2 program. Following the booster, there was a further reduction in weight loss, increased mean fruit and vegetable intake and greater mean daily PA of ≥30 min/day.

Consistent with the intervention paradigm of our prior studies, the intervention paradigm emphasizes increasing participants’ health knowledge and tying goals and challenges to the program curriculum (25–37). The incorporation of learning objectives distinguishes the intervention from other corporate wellness platforms relying primarily on promoting activities alone, without addressing health knowledge or the impact of health behaviors on outcomes. The durability of the outcomes and self-reported behavior during the year between the initial and booster interventions suggest that increasing health knowledge can be a critical factor in improving and sustaining health outcomes, which was a finding in our prior mediation analysis (31–33).

We did not perform the traditional health risk assessments (HRAs) used by many employers prior to either the Level-1 or Level-2 interventions. Our experience with use of HRAs and providing individualized interpretation was not useful as a behavior change intervention, even when accompanied with an itemized explanation and review of results (27, 29). Previous reviews of HRAs have found either no behavior change or minimal positive change, even with individualized feedback (65, 66). Even when HRAs were coupled with comprehensive, multiple individual coaching sessions using motivational interviewing, our prior tested team-based paradigm had more robust outcomes at a significantly lower cost (27, 29, 34). The Centers for Disease Control currently concludes that although HRAs “are widely used in workforce wellness programs … their use is not well understood, scientifically” (67). Furthermore, the Rand report found programs that identify employees with higher risks (e.g., HRAs), including those with hypertension, obesity, and use of tobacco, did not significantly reduce their healthcare costs (8).

Regardless of personal risk factors, the Level-1 and Level-2 interventions resulted in positive outcomes and acceptability among participants, which would lend support to the utility of a health promotion strategy addressing PA, mental health, nutrition, safety, sleep, and other healthy behaviors as components of a single comprehensive wellness offering for employees. This suggests that a broader approach aimed at all employees, may result in a cost-effective wellness strategy for organizations and employers, rather than segregating employees and instituting multiple interventions, each targeting specific health risks (8, 34).

### Limitations

Although the design was a prospective cohort analysis, there was no control group. A bias could have existed among employees completing the pre and post measures and those completing the pre-only measurements. Similarly, the longitudinal sample could be different than the initial pre and post sample, which could lead to erroneous conclusions. Also, although the pre- and post-test design can assist in making inferences on the effect of the intervention, causality cannot always be assigned.

Random assignment was not practical, due to the risk of contamination. A true control group not using another wellness initiative was not possible due to employee incentives offered by the employer. Despite these limitations, it did not appear that participants being assessed were different than other participants. Employees completing pre and post assessments were similar to those participants who completed the pre-measures only, with regard to biometrics and health behaviors (Tables 3 and 4). Likewise, employees completing the longitudinal analysis for physical measures and/or surveys were similar to those who completed the pre-assessment only. This suggests there was no differential dropout or selection bias based with regard to either pre-intervention behavior or biometric parameters.

To strengthen the evidence beyond statistical analysis, we have included the determination of effect size, which can help assess the magnitude of the program’s effect, without respect to sample size (47, 48). Effect sizes are in the medium category, providing more confidence in the relationship between the intervention and the observed effects (48). Although self-report assessment in can be influenced by social desirability, there is no reason to believe that this bias would differ across time points. Importantly, the positive outcomes were present for both objective and self-report measures, and across multiple health behaviors. Thus, despite potential drawbacks, the results of the wellness intervention had similar positive outcomes as our prior large randomized, controlled clinical trials (25–37).

## Conclusion

This study indicates that a research-tested, team-based, comprehensive health promotion and wellness program combined with a digital platform can be successfully translated to a diverse workplace setting. The positive objective biometric indices and self-reported outcomes among employee participants, indicate that a team-based structured program covering a wide range of health topics also may lead to enhanced employee cohesion, as well as produce medical cost savings as found in our previous intervention research on which this worksite intervention is based (27, 29, 34). In addition, the reduced stress and depression indices may predict future reductions in the prevalence of absenteeism and presenteeism (67–70).

## Conflict of Interest Statement

Healthy Team Healthy U is a program distributed by Provata Health, a technology transfer company of the Oregon Health & Science University (OHSU). OHSU and Drs. Linn Goldberg and Kerry Stephen Kuehl have a financial interest from the commercial sale of technologies described in this manuscript. This potential conflict of interest has been reviewed and managed by the OHSU Conflict of Interest in Research Committee.
